# *Abies holophylla* Leaf Essential Oil Alleviates Allergic Rhinitis Based on Network Pharmacology

**DOI:** 10.3390/pharmaceutics15041195

**Published:** 2023-04-09

**Authors:** Jae Yoon Chung, Nayoung Park, Mi Hye Kim, Woong Mo Yang

**Affiliations:** Department of Convergence Korean Medical Science, College of Korean Medicine, Kyung Hee University, 26 Kyungheedae-ro, Dongdaemun-gu, Seoul 02447, Republic of Korea

**Keywords:** allergic rhinitis, *Abies holophylla*, essential oil, inflammation, tight junction, network pharmacology

## Abstract

*Abies holophylla* is an evergreen coniferous species that has been widely used for treating pulmonary diseases and colds. Previous research has demonstrated the anti-inflammatory effect of *Abies* species and the anti-asthmatic activities of *Abies holophylla* leaf essential oil (AEO). As asthma and allergic rhinitis (AR) share pathophysiology and pharmacotherapeutic interventions, AEO inhalation can also ameliorate upper respiratory allergic diseases. This study explored the protective effects of AEO on AR with network pharmacological pathway prediction. The potential target pathways of AEO were analyzed by a network pharmacological approach. The BALB/c mice were sensitized by ovalbumin (OVA) and 10 μm particular matter (PM_10_) to induce allergic rhinitis. Aerosolized AEO 0.0003% and 0.03% were delivered by nebulizer for 5 min a day, 3 times a week for 7 weeks. Nasal symptoms (sneezing and rubbing), histopathological changes in nasal tissues, serum IgE, and zonula occludens-1 (ZO-1) expressions on nasal tissues were analyzed. After AR induction with OVA+PM_10_ and inhalation of AEO 0.0003% and 0.03% treatment, AEO significantly decreased allergic symptoms (sneezing and rubbing), hyperplasia of nasal epithelial thickness, goblet cell counts, and serum IgE level. The network analysis demonstrated that the possible molecular mechanism of AEO is highly associated with the IL-17 signaling pathway and tight junction. The target pathway of AEO was investigated in RPMI 2650 nasal epithelial cells. Treatment of AEO on PM_10_-treated nasal epithelial cells significantly reduced the production of inflammatory mediators related to the IL-17 signaling pathway, NF-κB, and the MAPK signaling pathway and prevented the reduction in TJ-related factors. When taken together, AEO inhalation may be considered as a potential treatment for AR by alleviating nasal inflammation and recovering the tight junction.

## 1. Introduction

Allergic rhinitis (AR) is a common inflammatory reaction in the nasal mucosal membrane caused by allergen inhalation, such as animal dander, pollen, or air pollution [1,2]. Epidemiological data showed that about 40% of the global population suffers from AR, with an increasing annual incidence [3,4]. Characteristic symptoms of AR include sneezing, rhinorrhea, itching, and nasal congestion [5]. These symptoms disrupt sleep and cause drowsiness during the day, significantly impairing the quality of life in both adults and children [6].

Exposure to environmental allergens provokes infiltration of allergen particles into the nasal epithelium with subsequent diffusion into the nasal mucosa. Especially particulate matter (PM) can aggravate the symptoms of AR [3]. In this process, allergens cleave tight junctions in nasal epithelium with their protease activities [7]. The damaged nasal epithelial cells produce cytokines and chemokines, inducing immune responses within and under the nasal epithelium [8,9]. These immune responses are mediated by IgE produced by B lymphocytes. IgE binds to the high-affinity receptor of mast cells, and re-exposure to allergen activates mast cells, inducing degranulation of mediators such as leukotrienes and histamines [10]. Leukotrienes increase mucus secretion and edema, causing nasal congestion [11]. Histamines induce sneezing and rubbing behavior by activating H1 receptors on sensory nerve endings [12].

Conventional treatments for AR are allergen avoidance and pharmacotherapy. Medications such as intranasal corticosteroids and antihistamines relieve characteristic symptoms of AR. However, the intranasal corticosteroid can cause throat irritation, burning, and epistaxis. Antihistamine administrations can cause nasal irritation, bitter aftertaste, and headache. Adverse effects of conventional medications made AR patients seek alternative therapies, which have fewer side effects [13,14,15]. For this reason, an increasing interest in herbal medicines that contain several natural compounds and have minimal or no undesirable side effects for treating various diseases is reported worldwide [16,17]. Herbal medicines also have a long history of beneficial effects in the alleviation of AR symptoms [18]. Herbal medicines such as *Flos magnolia*, *Angelicae dahuricae, Astragalus*, *Xanthii fructus*, and *Asarum* has been traditionally used for rhinitis treatments, and it is also used in rhinitis treatment these days [19].

Effective drug delivery into nasal tissues also plays an important role in AR management [20]. Nebulizer, an effective device for nasal drug delivery, delivers aerosolized medicine directly into the airway without hepatic first-pass metabolism [21]. This non-invasive drug administration requires a lower dosage of drugs compared to systemic treatments and has faster therapeutic effects [22]. As essential oils are lipophilic, they can be absorbed into eukaryotic cell membranes and directly affect nasal epithelial cells [23]. Essential oils extracted from herbs by distillation method also contain various volatile compounds such as monoterpenes and sesquiterpenes [24]. These various volatile compounds are reported to have anti-inflammatory and anti-microbial activities [25]. In addition, inhalation of essential oils has been used to treat diverse inflammatory diseases in the airway [26]. Therefore, AR treatment with essential oils and a nebulizer can effectively deliver various aerosolized pharmacological ingredients directly into the nasal mucosal membrane.

*Abies holophylla* is an evergreen coniferous species which is distributed widely in East Asia [27]. In traditional medicine, *Abies* species were widely used for treating pulmonary diseases, colds, and rheumatic diseases [28,29]. *Abies* species contain diverse sources of volatile bioactive compounds such as phenols, terpenoids, and sesquiterpenoids [30,31]. Previous research demonstrated that AEO had pharmacological effects, including anti-asthmatic effects, anti-inflammatory, and anti-fungal activities. Especially, inhalation of essential oils derived from *A. holophylla* had ameliorative effects on allergic asthma via suppressing inflammatory response in the lung, a lower respiratory tract [31,32]. Inhaled AEO has to pass through the upper respiratory system in order to reach and affect the lower respiratory tract. Based on the previous study, it is assumed that AEO inhalation can also ameliorate upper respiratory allergic rhinitis, as allergic asthma and allergic rhinitis share pathophysiology and pharmacotherapeutic interventions [33]. The biological activities and mechanisms underlying the anti-inflammatory effects of AEO inhalation on allergic rhinitis have not been studied.

Network pharmacology is a new strategy investigating the relationship among diseases, drugs, and targets systemically [34]. Increasing evidence supports that network pharmacology is an effective tool for investigating molecular mechanisms of herbs that contain complex multi-compounds [35]. Here, the network pharmacologic approaches were applied to systematically investigate the pharmacologic effects and potential targets of AEO on allergic rhinitis. Based on network pharmacological prediction, the anti-inflammatory and tight junction recovery effects of AEO inhalation on allergic rhinitis were demonstrated by in vivo and in vitro experiments. In this study, we used OVA and PM_10_-induced allergic rhinitis mice model to investigate whether AEO could inhibit nasal behavior and inflammation in the condition of PM exposure-exacerbated allergic responses.

## 2. Materials and Methods

### 2.1. Network Construction and Prediction of Genes Associated with Allergic Rhinitis

The AEO network was constructed with AEO compounds. 5 compounds of AEO, bicyclo [2.2.1]heptan-2-ol, Camphene, dl-limonene, α-Pinene, δ3-Carene (PubChem CID: 79028, 6616, 22311, 6654, and 26049) was gathered base on reference [32]. Genes related to AEO compounds were organized with PubChem (https://pubchem.ncbi.nlm.nih.gov/) chemical gene co-occurrence accessed on 8 June 2022. After eliminating duplicates, a protein-protein interaction network (PPIN) was constructed with 172 genes related to AEO using Cytoscape sting App. AR-related genes were gathered using GeneCards (http://www.genecards.org/ (accessed on 8 June 2022)) with the keyword “Allergic rhinitis.” Common genes of the AEO network and AR-related gene set were organized. All collected databases were provided as a Supplementary File S1.

### 2.2. KEGG Functional Enrichment Analysis

Biological pathways related to the AEO network were investigated with Cytoscape functional enrichment analysis. The pathways and targets of AEO were analyzed and categorized by the KEGG pathway 2021 human and Gene Ontology (GO) Process. AR-related KEGG pathways and GO process were organized, and cut-off criteria were *p*-value < 0.05.

### 2.3. Preparation of AEO

The fresh Leaf of *Abies holophylla* was collected from PyeongChang, Korea. 100 g *Abies holophylla* leaf was submitted to steam distillation apparatus. Distillation continued for 5 h at 100 °C with 1 L distilled water. A total of 0.8 mL of AEO was obtained and kept at 4 °C before experiments.

### 2.4. Animal Models and Drug Administration

BALB/c mice (5 weeks old, female) were obtained from DBL Co., Ltd. (Eumseong-gun, Republic of Korea). The mice were kept in controlled room humidity (45–55% humidity), temperature (20–24 °C), and 12 h light/dark cycle. The approval of animal experiment protocols (KHUASP(SE)-20-363) was attained by Kyung Hee University Institutional Animal Ethics Committee in Korea. The animal experimental design was followed by a previous study [31]. After acclimatization for a week, mice were randomly divided into 5 groups (*n* = 7); NOR, the normal saline-sensitized group; OVA+PM_10_, the ovalbumin, and 10 μm particular matter treated group; DEX, dexamethasone-treated group; AEO 0.0003% and AEO 0.03%, AEO 0.0003% *v*/*v* and 0.03% *v*/*v* treated group, respectively. On days 1 to 47, the DEX, AEO 0.0003%, and AEO 0.03% group inhaled aerosolized DEX and AEO with a nebulizer, 5 min per time and 3 times per week. The concentration of DEX was 0.06% *w*/*v* (2 mg/kg). The NOR and OVA+PM_10_ groups inhaled saline. The spray amount was 1 mL/min. On days 21, 28, and 35, all experimental groups except NOR were injected with 10 mg ovalbumin and 500 mg aluminum hydroxide dissolved in 0.1 mL saline intraperitoneally. NOR group was injected with 0.1 mL saline. From day 42 to 44, all groups except the NOR group received instillation of 100 μg PM_10_ and 1 mg OVA in 50 μL saline intranasally. All Mice were sacrificed on day 49.

### 2.5. Histopathological Examination

Nasal tissues were collected, dissected, and fixed in 10% formalin for a day. Fixed tissues were dehydrated with ethanol and xylene, embedded in paraffin, then sectioned with a microtome at 5 μm thickness. The epithelial thickness was measured after staining tissues with hematoxylin and eosin (H&E). Goblet cells were counted after staining sections with periodic acid-schiff (PAS). Specimens were stained with Toluidine blue to visualize the mast cell hyperplasia in the nasal tissues. We randomly selected a total of three images for each unit of each group. For epithelial thickness, we measured the thickness of the epithelium at two randomly selected points in each image. We also randomly selected images for goblet cell and mast cell counting and measured their numbers by counting stained cells.

### 2.6. Nasal Sneezing and Rubbing Behavior

To evaluate allergen-induced nasal symptoms, sneezing, and rubbing frequency were counted. Each time the mice touched or rubbed the area around the nose using a forepaw was counted as one rubbing event. Five blinded observers counted the frequency of sneezing and rubbing times 20 min after the last OVA exposure. The actions were recorded by video camera, and the average score was analyzed.

### 2.7. Serum Levels of IgE

Blood samples were obtained from the mice via cardiac puncture. The collected samples were centrifuged (17,000 rpm, 30 min at 4 °C) and serum was collected. The IgE levels on serum were measured with a commercial ELISA kit (BD Biosciences, San Diego, CA, USA), following the manufacturer’s recommendations.

### 2.8. Cell Counts for NALF

The NALF was collected after sacrifice by gently perfusing 1 mL sterile saline solution from the choanae to the nostril. The cells in NALF were centrifuged. The inflammatory cell numbers (neutrophils, eosinophils, macrophages, and lymphocytes) in NALF were measured with a hemocytometer based on staining characteristics and cellular morphology after Wright-Giemsa staining. The total cell numbers of NALF were summed from the numbers of neutrophils, eosinophils, macrophages, and lymphocytes.

### 2.9. Immunohistochemistry

The sectioned nasal tissues were deparaffinized and treated with 3% H_2_O_2_ for 30 min to block endogenous peroxidase. Samples were incubated with primary antibody ZO-1 (1:500, Santa Cruz Technology Inc., Dallas, TX, USA) at room temperature overnight, followed by incubation with mouse-absorbed biotinylated goat anti-rat IgG (1:200, Vector laboratories Inc., Newark, CA, USA) for 1 h at room temperature. After PBS washing, samples were incubated with Elite ABC Kit (Vector laboratories, CA, USA), and then peroxidase conjugates were subsequently visualized with diaminobenzidine (Sigma-Aldrich; Merck Millipore, Darmstadt, Germany) solution. The sections were counterstained with hematoxylin.

### 2.10. Cell Culture

RPMI 2650, a nasal mucosal cell line, was obtained from the Korean Cell Line Bank (Seoul, Republic of Korea). RPMI 2650 was cultured in a 5% CO_2_ atmosphere at 37 °C. The culture medium was 10% fetal bovine serum (FBS, Corning, Somerville, MA, USA) and 1% penicillin/streptomycin containing RPMI 1640 (Corning, USA).

### 2.11. MTT Cytotoxicity Assay

RPMI 2650 cells were detached with Trypsin-EDTA solution and replated onto 96-well plates at 3 × 10^4^ cells/100 µL per well. Various concentrations (10^−4^, 10^−3^, and 10^−2^% *v*/*v*) of AEO were added to each well and incubated for 24 h. After incubation, MTT solution (2 mg/mL in PBS) was added and incubated at 37 °C for 2 h. The 100 μL DMSO (Sigma Aldrich, Seoul, Republic of Korea) were treated to solubilize formazan crystals formed. The absorbance values of the sample were read using an ELISA plate reader at 570 nm.

### 2.12. AEO Treatment of RPMI2650

RPMI 2650 cells were seeded at 1 × 10^6^ cells/2 mL per well into 6-well plates. RPMI 2650 cells were incubated with 100 μg/mL of PM_10_ (NIST, Gaithersburg, MD, USA) in the presence of 10^−4^, 10^−3^, and 10^−2^% *v*/*v* of AEO and 1 μM DEX for 24 h.

### 2.13. Reverse Transcriptase Polymerase Chain Reaction (RT-PCR)

Total RNA was extracted from the cells by TRIZOL reagent (Invitrogen, Carlsbad, CA, USA). Complementary DNA was synthesized with a Maxime RT premix kit (Invitrogen, Carlsbad, CA, USA). 1000 ng of cDNA was synthesized at 45 °C for 60 min and at 95 °C for 5 min. RT-PCR was used to analyze mRNA expression of the IL-17 signaling pathway and tight junction-related factors with i-StarTaq™ (Invitrogen, Waltham, MA, USA). Glyceraldehyde-3-phosphate dehydrogenase (GAPDH) was used for housekeeping gene control, and all primer sequences were described in Table 1. The amplified samples were verified on 2% agarose gel using gel electrophoresis. The relative density of bands was measured by the Image J program (v1.4.3.x., U. S. National Institutes of Health, Bethesda, MD, USA).

### 2.14. Western Blot Analysis

RPMI 2650 cells were lysed in cold RIPA buffer (Thermo Scientific, Rockford, IL, USA). The concentrations of protein were analyzed by Bradford protein assay (Bio-Rad, Hercules, CA, USA). The cytosolic and nuclear fractions were isolated by TE-PER Nuclear and Cytoplasmic Extraction Reagents (Thermo Scientific). The proteins were separated by SDS-PAGE in accordance with protein size and electrophoretically transferred to a polyvinylidene difluoride (PVDF) membrane. The PVDF membranes were incubated in blocking TBS-T buffer containing 3% bovine serum albumin for 1h at room temperature. The membranes were treated with the corresponding primary antibodies; Tumor necrosis factor receptor associated factor (TRAF6)(1:1000, Santa Cruz), ERK (1:1000, Cell Signaling Technology, Danvers, MA, USA), phospho-ERK (1:1000, Cell Signaling Technology), JNK (1:1000, Cell Signaling Technology), phospho-JNK (1:1000, Santa Cruz), p38 (1:1000, Cell Signaling Technology), phospho-p38 (1:1000, Santa Cruz), NF-κB p65 (1:1000, Cell Signaling Technology), Lamin-B (1:1000, Santa Cruz), β-actin (1:2000, Santa Cruz), an inhibitor of nuclear factor kappa B (IκB-α) (1:1000, Cell Signaling Technology), phospho-IκB-α (1:1000, Santa Cruz), claudin-1 (1:1000, Santa Cruz), ZO-1 (1:1000, Santa Cruz), occludin (1:1000, Santa Cruz), junctional adhesion molecules (JAM)-A (1:1000, Abcam, Cambridge, UK), GAPDH (1:2000, Santa Cruz) for overnight at 4 °C. Then the membranes were treated with secondary antibodies (1:3000, cell signaling) for 1 h at room temperature. The signals of proteins were detected by enhanced chemiluminescence (Amersham, Uppsala, Sweden), and the density of bands was measured by the Image J program. The relative protein levels were determined by comparing the protein expression level of each sample treatment group with the control group, not treated with PM, DEX, and AEO.

### 2.15. Statistical Analysis

The statistical analyses of data were proceeded using Prism 5 (GraphPad Software, Inc., La Jolla, CA, USA). A probability value of *p* < 0.05 was considered statistically significant. Data were expressed with a means ± standard error of the mean (SEM). The comparison between groups was made using one-way ANOVA followed by Tukey.

## 3. Results

### 3.1. AEO Network and Shared Targets with Allergic Rhinitis

**Figure 1:**
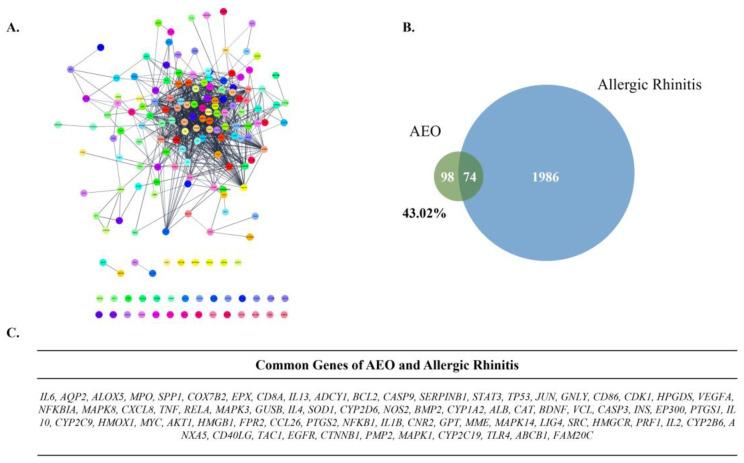
Network analysis of AEO. (**A**) PPIN of AEO. (**B**) Venn-diagram of AEO and Allergic Rhinitis gene set. (**C**) Common genes of AEO and allergic rhinitis gene set.

The compounds of the AEO network, *bicyclo [2.2.1]heptan-2-ol, Camphene, dl-limonene, α-Pinene,* and *δ3-Carene* had 7, 100, 100, 100, 100, and 99 co-efficient genes, respectively. The PPIN of AEO was composed of 172 nodes and 876 edges (Figure 1A). The number of common genes between the AEO network and the allergic rhinitis gene set was 74. The identical genes of AEO matched with the allergic rhinitis gene set was 43.02% of the total target genes of AEO (Figure 1B). The common genes of allergic rhinitis and the AEO network were organized (Figure 1C).

### 3.2. KEGG and GO Enrichment Analysis of AEO

To predict the significant target pathway of AEO on allergic rhinitis, functional enrichment analysis results of AEO were organized, and allergic rhinitis-related pathways were sorted (Table 2). In KEGG pathways, the ‘IL-17 signaling pathway’ and ‘Tight junction’ were revealed to participate in the underlying mechanism of AEO. In the Go process, ‘Cellular response to chemical stimulus’ and ‘Response to external stimulus’ were enriched with *p*-value < 0.05.

### 3.3. Histological Changes of Nasal Tissues

**Figure 2:**
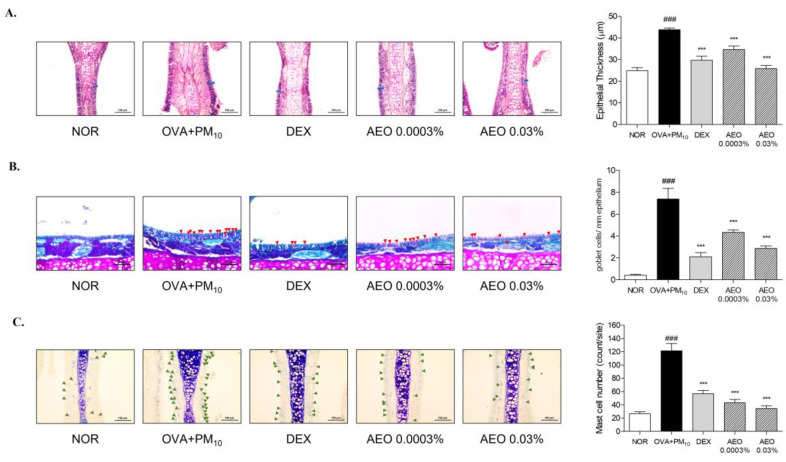
AEO ameliorated histological features presented in allergic rhinitis-like nasal epithelium of OVA+PM_10_-induced allergic rhinitis mice. Nasal epithelial tissues were stained with H&E. Blue bars indicate the nasal epithelial thickness (magnification × 200) (**A**). Goblet cells were stained with PAS indicated as a red arrowhead(magnification × 400) (**B**). Mast cells were stained with toluidine blue, indicated as a green arrowhead (magnification × 200) (**C**). The data are presented as the means ± standard error of the mean. ### *p* < 0.001 compared to the NOR group. *** *p* < 0.001 compared to OVA+PM_10_ group.

H&E staining showed a significant increase in epithelial thickness in OVA+PM_10_-exposed mice than NOR group. AEO 0.0003% and 0.03% groups showed markedly decreased epithelial thickness by 28.23% and 41.06% than the OVA+PM_10_-treated group (Figure 2A). The PAS staining displayed that the number of goblet cells significantly increased in the OVA+PM_10_-exposed mice than NOR group. AEO 0.0003% and 0.03% treated group significantly downregulated goblet cell counts by 41.24% and 61.15% to the OVA+PM_10_-treated mice (Figure 2B). Toluidine blue staining of nasal tissues showed that the number of mast cells was significantly higher in the OVA+PM_10_-exposed group compared to the NOR group. The AEO 0.0003% and 0.03% groups showed markedly downregulated mast cell numbers by 64.47% and 71.78% compared to the OVA+PM_10_-treated mice (Figure 2C). The DEX-treated group provided significant protection against nasal tissue damage.

### 3.4. Effects on Nasal Sneezing and Rubbing Behavior

**Figure 3:**
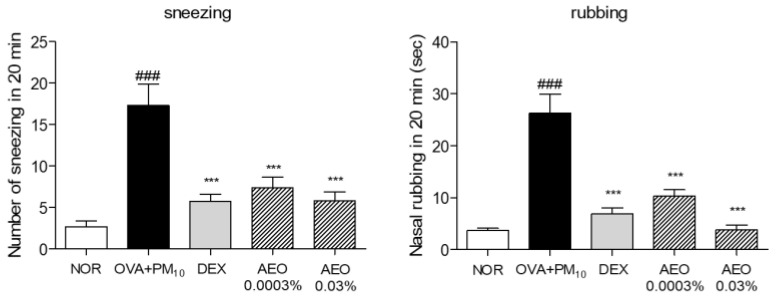
AEO decreased the frequency of sneezing and rubbing behavior in OVA+PM_10_-induced allergic rhinitis mice. The number of sneezing and rubbing was counted for 20 min through watching by a fiver observer and recorded with the video. The data are presented as the means ± standard error of the mean. ### *p* < 0.001 compared to the NOR group. *** *p* < 0.001 compared to OVA+PM_10_ group.

After the last intranasal administration, the frequency of sneezing and rubbing significantly increased in the OVA+PM_10_-treated group compared to the NOR group. AEO 0.0003% and 0.03% groups showed a significantly reduced incidence of sneezing by 57.59% and 66.45% compared to the OVA+PM_10_-treated group. The AEO 0.0003% and 0.03% treated group showed a markedly decreased frequency of rubbing by 60.65% and 85.58% compared to the OVA+PM_10_-treated group. The DEX-treated group also showed a significantly decreased incidence of sneezing and rubbing behavior by 66.94% and 73.97% compared to the OVA+PM_10_-treated group (Figure 3).

### 3.5. Effects on Serum IgE Level

**Figure 4:**
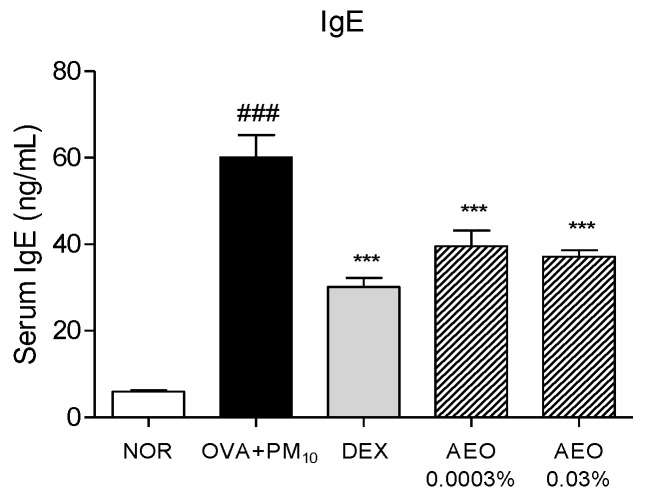
AEO reduced serum IgE levels in OVA+PM_10_-induced allergic rhinitis mice. The data are presented as the means ± standard error of the mean. ### *p* < 0.001 compared to the NOR group. *** *p* < 0.001 compared to OVA+PM_10_ group.

Increased serum IgE levels were found in the OVA+PM_10_-treated group compared to the NOR group. The AEO 0.0003% and 0.03% groups showed significantly downregulated serum IgE levels by 34.19% and 38.25%. The serum IgE levels of the DEX-treated group significantly decreased compared to the OVA+PM_10_-treated group (Figure 4).

### 3.6. Effects on Infiltration of Differential Inflammatory Cells in NALF

**Figure 5:**
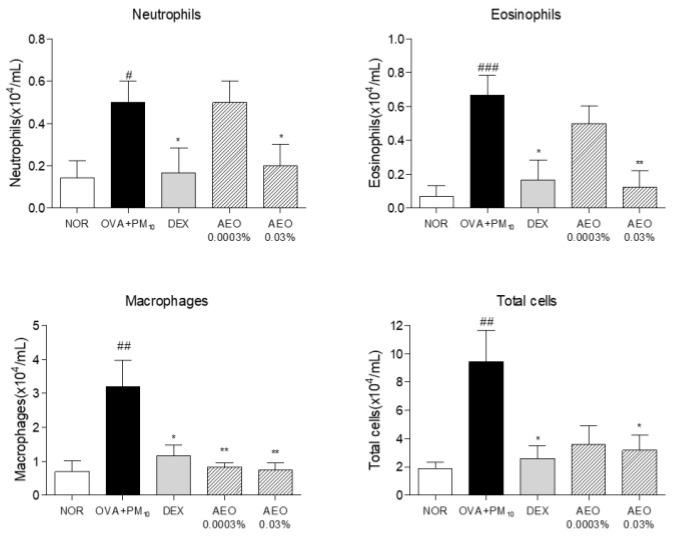
AEO attenuated neutrophils, eosinophils, macrophages, and total cells in NALF of OVA+PM_10_-induced allergic rhinitis mice. The number of cells in NALF was counted based on the morphological features of the cells. The data are presented as the means ± standard error of the mean. # *p* < 0.05, ## *p* < 0.01, ### *p* < 0.001 compared to NOR group. * *p* < 0.05, ** *p* < 0.01 compared to OVA+PM_10_ group.

The OVA+PM_10_-treated group had an increased number of neutrophils, eosinophils, macrophages, and total cell (neutrophils, eosinophils, macrophages, and lymphocyte) numbers in NALF compared to the NOR group. The AEO 0.03% treatments decreased neutrophil counts by 60% compared to the OVA+PM_10_-treated group. The AEO 0.03 % treatments decreased the number of eosinophils by 81.25% compared to the OVA+PM_10_-treated group. The AEO 0.0003% and 0.03% treated group showed a significantly reduced number of macrophages by 73.06% and 76.56% compared to the OVA+PM_10_-treated group. The AEO 0.03% treatments decreased total cell counts by 66.31% compared to the OVA+PM_10_-treated group. The numbers of inflammatory cell numbers (neutrophils, eosinophils, macrophages, and total cells) in DEX treated group were significantly reduced by 66.67%, 75%, 63.54%, and 72.37%, respectively, compared to the OVA+PM_10_-treated group (Figure 5). However, the number of lymphocytes was not changed by AEO treatment.

### 3.7. Cytotoxicity of AEO

The cytotoxicity of AEO on RPMI2650 was determined by the MTT assay. The cell viability was not reduced significantly by various concentrations of AEO (10^−4^, 10^−3^, and 10^−2^% *v*/*v*) (Supplementary File S2).

### 3.8. Effects on IL-17 Signaling Pathway Related Factors in the PM_10_-Treated Nasal Epithelial Cells

**Figure 6:**
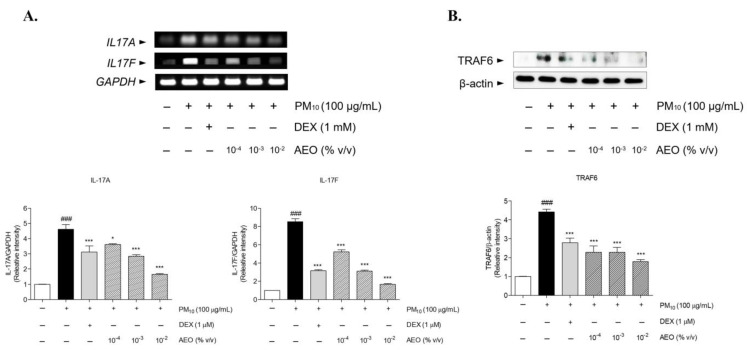
AEO decreased mRNA expression levels of *IL17A*/*F* in PM_10_-induced human nasal epithelial cells (**A**). The mRNA levels of *IL17A*/*F* were analyzed by RT-PCR. AEO decreases protein expression levels of TRAF6 in PM_10_-induced human nasal epithelial cells (**B**). The protein levels of TRAF6 were analyzed by western blot assay. The data are presented as the means ± standard error of the mean. ### *p* < 0.001 compared to the NOR group. * *p* < 0.05, *** *p* < 0.001 compared to PM_10_-treated group.

The mRNA expression of *IL17A* and *IL17F* increased in PM_10_-treated human epithelial cells. The AEO (10^−4^, 10^−3^, and 10^−2^% *v*/*v*) treatments markedly downregulated the increased mRNA levels of *IL17A* by 21.76%, 38.53%, and 64.13%. The AEO (0.001, 0.01, and 0.1 μL/mL) treatments significantly reduced the *IL17F* mRNA expression by 38.91%, 63.61%, and 80.49%, respectively. The 1 μM DEX treatments significantly reduced *IL17A* and *IL17F* mRNA levels by 32.48% and 62.93% (Figure 6A). The TRAF6 protein expression was upregulated after PM_10_-treatment. Treatment of AEO (10^−4^, 10^−3^, and 10^−2^% *v*/*v*) significantly reduced TRAF6 protein expression by 48.32%, 48.39%, and 59.67%. The 1 μM DEX treatment decreased TRAF6 protein expression by 36.98% (Figure 6B).

### 3.9. Effects on the MAPK-Related Factors in PM_10_-Treated Nasal Epithelial Cells

**Figure 7:**
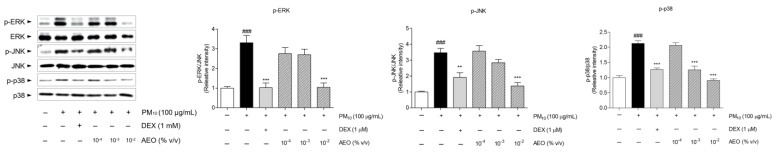
AEO reduced expression levels of p-ERK, p-JNK, and p-p38 in PM_10_-induced human nasal epithelial cells. The protein levels of p-ERK, p-JNK, and p-p38 were analyzed by western blot assay. The data are presented as the means ± standard error of the mean. ### *p* < 0.001 compared to the NOR group. ** *p* < 0.01, *** *p* < 0.001 compared to PM_10_-treated group.

The phosphorylation of MAPK-related factors, extracellular signal-regulated kinase (ERK), c-Jun N-terminal kinase (JNK), and p38 significantly increased in the PM_10_-treated group. The expressions of p-ERK markedly decreased in AEO 10^−2^% *v*/*v* treated groups by 68.56%. The levels of p-JNK significantly reduced in AEO 10^−2^% *v*/*v* treated groups by 60.33%. The phosphorylation of p38 significantly decreased in AEO (10^−3^ and 10^−2^% *v*/*v*) treated groups by 40.98% and 57.41%. The treatment of 1 μM DEX regulated phosphorylation of ERK, JNK, and p38 by 69.05%, 44.89%, and 40.2%, respectively (Figure 7).

### 3.10. Effects on the NF-κB-Related Factors in PM_10_-Treated Nasal Epithelial Cells

**Figure 8:**
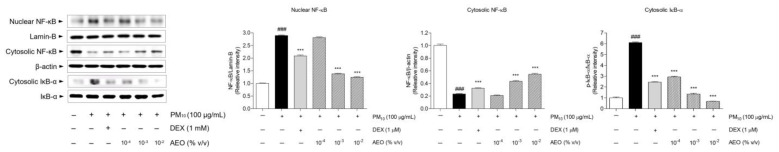
AEO regulated expression levels of NF-κB signaling pathways on PM_10_-induced human nasal epithelial cells. The protein levels of nuclear NF-κB, cytosolic NF-κB, and cytosolic IκB-α were analyzed by western blot assay. The data are presented as the means ± standard error of the mean. ### *p* < 0.001 compared to the NOR group. *** *p* < 0.001 compared to PM_10_-treated group.

The expression levels of NF-κB signaling pathway-related factors, nuclear NF-κB and phosphorylated cytosolic IκB-α markedly increased, and cytosolic NF-κB decreased in the PM_10_-treated group. The AEO (10^−3^ and 10^−2^% *v*/*v*) treatments significantly downregulated expression of nuclear NF-κB by 52.25% and 57.04%. Expressions of cytosolic NF-κB significantly increased in the AEO (10^−3^ and 10^−2^% *v*/*v*) treated groups by 87.11% and 134.83%. The AEO (10^−4^, 10^−3^, and 10^−2^% *v*/*v*) treatments markedly reduced expressions of phosphorylated cytosolic IκB-α by 51.98%, 77.97%, and 88.85%. The 1 μM DEX treatment reduced expressions of nuclear NF-κB and cytosolic IκB-α and increased cytosolic NF-κB by 27.72%, 59.93%, and 39.84%, respectively (Figure 8).

### 3.11. Effects on ZO-1 Protein Expressions on Nasal Tissues

**Figure 9:**
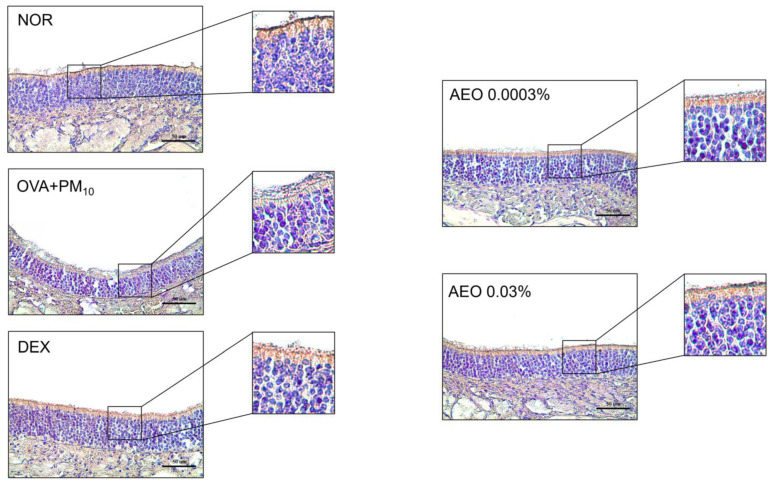
AEO increased expression of ZO-1 in OVA+PM_10_-induced allergic rhinitis mice. The expressions of ZO-1 were analyzed by immunohistochemistry. Tissue sections were observed under a microscope (magnification 400×).

The expression levels of ZO-1 were evaluated with immunohistochemistry. The downregulated expression of ZO-1 (black box) was found in the OVA+PM_10_ group compared to NOR. The ZO-1 expression in the nasal epithelial tissues was found to be restored in AEO 0.003% and 0.03% groups (Figure 9).

### 3.12. Effects on Tight Junction-Related Factors in the PM_10_-Treated Nasal Epithelial Cells

**Figure 10:**
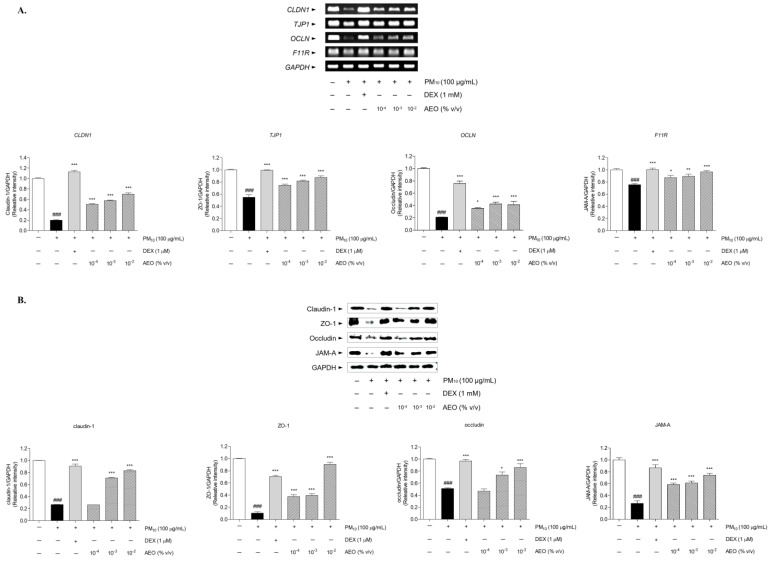
Expressions of tight junction-related factors in PM_10_-treated nasal epithelial cells. AEO increased mRNA expression levels of *CLD1* (Claudin-1), *TJP1* (ZO-1), *OCLN* (Occludin), and *F11R* (JAM-A) in PM_10_-induced human nasal epithelial cells (**A**). AEO increased protein expressions of ZO-1, Claudin-1, Occludin, and JAM-A PM_10_-induced human nasal epithelial cells (**B**). The data are presented as the mean ± means ± standard error of the mean. ### *p* < 0.001 compared to the NOR group. * *p* < 0.05, ** *p* < 0.01, *** *p* < 0.001 compared to PM_10_-treated group.

PM_10_ treatment to nasal epithelial cells significantly decreased mRNA expression levels of tight junction-related mRNA compared to the NOR group. The mRNA expression of *CLDN1* increased by 152.25%, 189.81%, and 250.01% in the AEO (10^−4^, 10^−3^, and 10^−2^% *v*/*v*) treated groups compared to PM_10_ treated group. The AEO (10^−4^, 10^−3^, and 10^−2^% *v*/*v*) treatments significantly upregulated mRNA expression of *TJP1* by 35.65%, 48.66%, and 58.99% compared to the PM_10_ treated group. Moreover, the mRNA expressions of *OCLN* in AEO (10^−4^, 10^−3^, and 10^−2^% *v*/*v*) treated groups markedly increased by 68.98%, 101.97%, and 96.59% compared to the PM_10_ treated group. The treatment of AEO (10^−4^, 10^−3^, and 10^−2^% *v*/*v*) increased mRNA expressions of *F11R* by 15.32%, 18.01%, and 27.74% than the PM_10_ treated group. The 1 μM DEX treatment increased mRNA expressions of *CLDN1*, *TJP1*, *OCLN*, and *F11R* by 463.97%, 80.6 %, 260.89%, and 32.51% compared to the PM_10_-treated group, respectively (Figure 10A).

The protein expression levels of tight junction-related factors, claudin-1, ZO-1, occludin, and JAM-A, significantly decreased in the PM_10_-treated group. Treatments of AEO (10^−3^ and 10^−2^% *v*/*v*) significantly increased protein expressions of claudin-1 by 163.37% and 209.38% compared to the PM_10_-treated group. The protein expressions of ZO-1 were significantly upregulated in AEO (10^−4^, 10^−3,^ and 10^−2^% *v*/*v*) treated group by 256.37%, 275.59%, and 759.4% compared to the PM_10_-treated group. The AEO (10^−3^ and 10^−2^% *v*/*v*) treatment increased protein expressions of occludin by 44.09% and 68.58% compared to the PM_10_-treated group. Moreover, AEO (10^−4^, 10^−3^, and 10^−2^% *v*/*v*) treatment increased protein expressions of JAM-A by 106.36%, 115.85%, and 131.15% compared to the PM_10_-treated group. The protein expressions of claudin-1, ZO-1, occludin, and JAM-A in DEX treated group increased by 236.65%, 569.88 %, 90.05%, and 224.18% compared to the PM_10_-treated group, respectively (Figure 10B).

## 4. Discussion

AR is an inflammatory disease in the upper airway with characteristic features such as inflammatory cell recruitment, increased inflammatory mediators, and epithelial cell destruction [36,37]. Exposure to allergens brings metaplasia of squamous cells in the nasal mucosa, causing nasal obstruction [38]. Allergen exposures also increase the number of goblet cells in nasal tissues, resulting in mucus hypersecretion [38,39,40,41]. The present study demonstrated that AEO treatment significantly reduced nasal epithelial thickness and goblet cell counts in OVA+PM_10_-treated mice. These results demonstrate that AEO can relieve nasal congestion and rhinorrhea, which are characteristic symptoms of AR in mice models.

Mast cells are an important effector in eosinophilic inflammation induction and maintenance. Mast cells are activated through crosslinking with antigen-specific IgE in allergen-exposed conditions [42]. Activated mast cells release granules containing inflammatory cytokines, chemokines, and histamines, which initiate sneezing and rubbing behavior and increase eosinophils and inflammatory cells in NALF [43]. Eosinophils cause the destruction of airway epithelium and promote AR development with inflammatory mediators produced by mast cells [37,44,45]. In this study, AEO inhalation significantly downregulated mast cell infiltrations, and serum IgE levels, along with sneezing and rubbing behavior, eosinophils, and inflammatory cell counts in NALF. These findings indicate that AEO can effectively mediate mast cell mediated-inflammatory responses of AR in mice models.

Network pharmacology reveals intervention mechanisms and therapeutic effects of herbal medicines on diseases from the PPIN network and the interaction relationship between herb medicines, targets, and diseases [46]. Network pharmacology systemically revealed a correlation between the AEO network and the AR gene set. The 74 genes among 172 AEO network-constructing genes were identical to the AR gene set, which matched 43.02% of AEO network genes. The PPIN network of AEO was further analyzed with Cytoscape to explore allergic rhinitis-related pathways. The network analysis using the AEO compound network and AR gene set demonstrated that the possible molecular mechanism of AEO was highly associated with the IL-17 signaling pathway and tight junction.

IL-17 is a proinflammatory cytokine that is associated with the clinical symptoms of AR. IL-17 expression in nasal mucosa regulates innate immunity by inducing the production of inflammatory mediators [47,48]. Recent research on AR found that IL-17 increases eosinophil accumulation and serum IgE production, triggering allergic reactions [8,49]. The upregulation of IL-17 can increase airway permeability to allergens, enhance airway hyperactivity, and increase mucous production of airway epithelial cells [50,51]. In the present research, the AEO treatments markedly decreased the elevated levels of *IL17A* and *IL17F* in PM_10_-treated nasal epithelial cells. The downstream mediator of IL-17, TRAF6, is necessary for IL-17 mediated NF-κB and MAPK signaling pathways activation [52,53,54]. Recruitment of TRAF6 triggers activation of NF-κB pathways, which increases expressions of cytosolic IκB-α and nuclear NF-κB [55,56]. Furthermore, activation of the NF-κB pathway triggers transcriptions of inflammatory cytokines and chemokines, resulting in inflammatory responses, such as the recruitment of eosinophils and neutrophils [57]. MAPK pathways, which regulate the cellular response to cytokines, include ERK, p38, and JNK [58]. The phosphorylation of ERK, p38, and JNK induces the transcription of inflammatory mediators in allergic inflammation in AR [59]. In this study, AEO treatments significantly regulated expressions of TRAF6, NF-κB pathway-related factors, and phosphorylation of ERK, JNK, and p38 in PM_10_-treated nasal epithelial cells. Together with in vivo results, these results suggest that AEO treatments can effectively suppress inflammatory reactions in nasal epithelial cells by regulating IL-17 mediated NF-κB and MAPK signaling pathways. Nevertheless, this study has limitations in that molecules of the IL-17 signaling pathway regarded as one of the main functional pathways of AEO was not investigated in allergic rhinitis mice. Generally, it is considered that it is difficult to obtain a sufficient sample from mice and detect low-abundance molecules. Additionally, the heterogeneity of nasal tissue can be one of the reasons to make it is hard to analyze the molecules in animal tissues.

TJ exists in the apical region of the airway epithelium and provides initial protection against allergens, pathogens, and chemical stress [60]. The maintenance of TJ in nasal epithelial cells depends on expressions of TJ comprising transmembrane proteins, such as zonula occludes, occludin, claudin, and JAM [7,61]. These TJ proteins, including ZO-1, claudin, occludin, and JAM-A, form complex connections between nasal epithelial cells, blocking the passage of allergens into the subepithelial area [62]. ZO-1, an adaptor protein, and regulator of TJ assembly interacts with the C-terminal domains of claudin, occludin, and JAM-A to form cell-to-cell adhesion [36]. Claudin-1, a barrier claudin expressed in most tissues ubiquitously, is essential for the stability of the epithelial barrier [63]. The increased expressions of claudin-1 tighten the epithelium and decrease the permeability of the epithelium [64]. Occludin acts as tightening component of TJ, and its expression modulates airway epithelial permeability [65]. JAM-A, an immunoglobulin superfamily protein located at epithelial junctions, regulates tight junction assembly [66]. JAM-A localization in TJ regulates epithelial barrier function by interaction with ZO-1 [67]. The downregulation of TJ proteins during AR development impairs nasal epithelial barrier function, leading to increased pre-cellular permeability, external antigen invasion, and immune cell activation [68]. Treatment of PM_10_ on nasal epithelial cells causes TJ disruption by downregulating ZO-1, claudin-1, and occludin [69]. Recent research indicated that recovery of decreased TJ levels could provide alternative therapeutic strategies in AR treatments [70,71,72]. In the present research, AEO treatment significantly prevented the downregulated expressions of protein of ZO-1 in OVA+PM_10_ treated-nasal epithelial tissues and mRNA and protein expressions of ZO-1, claudin-1, occludin, and JAM-A on PM_10_-treated nasal epithelial cells. To sum up, the results of this study indicate that AEO recovers TJ functions and becomes alternative therapeutic agent in AR treatment.

## 5. Conclusions

In summary, this study demonstrates that inhalation of AEO can ameliorate allergic rhinitis by regulating inflammatory reactions and restoring tight junction integrity. AEO treatment ameliorated AR by decreasing nasal epithelial thickness, hyperplasia of goblet cells, allergic symptoms (sneezing and rubbing), serum IgE, inflammatory cells infiltration, and ZO-1 expression in nasal epithelial tissues in OVA+PM_10_-induced AR mice. The underlying mechanisms of AEO were predicted by network analysis. The AEO treatment downregulated NF-κB and MAPK signaling pathways by inhibiting the IL-17 signaling pathway in nasal epithelial cells. The AEO treatment also prevented tight junction destruction by upregulating expressions of ZO-1, occludin, claudin-1, and JAM-A in nasal epithelial cells. Overall, the findings of this study suggest that AEO could be a potential alternative treatment for AR.

## Figures and Tables

**Table 1:** Primers used for RT-PCR of IL-17 signaling pathway and tight junction-related factors.

Gene	Forward Primer	Reverse Primer
*IL17A*	TCCAGAAGGCCCTCAGACTA	AGCATCTTCTCGACCCTGAA
*IL17F*	GTGCCAGGAGGTAGTATGAAGC	ATGTCTTCCTTTCCTTGAGCATT
*CLDN1* (Claudin-1)	CTTCATTCTCGCCTTCCT	TGACAGCCATCCTCATCTT
*TJP1* (ZO-1)	GGAGAGGTGTTTCGTGTTGT	ACTGCTCAGCCCTGTTCTTA
*OCLN* (Occludin)	TATGCCCTCTGCAACCAA	CACCGCTGCTGTAACGAG
*F11R* (JAM-A)	CTTCGATCCTGTGTCAGCTT	TCTATAGGCGAACCAGATGC
*GAPDH*	CCATCACCATCTTCCAGGAG	CCTGCTTCACCACCTTCTTG

**Table 2:** AEO target pathway based on KEGG 2021 human pathway and GO process.

Category	Description	*p*-Value	Background Genes	Common Genes
KEGG Pathways	IL-17 signaling pathway	3.39 × 10^−18^	92	18
	Tight junction	0.003	156	5
GO process	Cellular response to chemical stimulus	2.07 × 10^−37^	2919	99
	Response to external stimulus	2.94 × 10^−25^	2310	75

## Data Availability

The data used to support the findings of this study are available from the corresponding author upon reasonable request.

## Supplementary Materials

The following supporting information can be downloaded at: https://www.mdpi.com/article/10.3390/pharmaceutics15041195/s1, File S1: The collected database from TM-MC, Pubchem and GeneCards. File S2: Cell viability of RPMI 2650 cells by AEO (0.001, 0.01, and 0.1 μL/mL) treatments.
