# Supplementary figures and images for: Abies holophylla Leaf Essential Oil Alleviates Allergic Rhinitis Based on Network Pharmacology
Source: Pharmaceutics. 2023 Apr 9;15(4):1195. doi: 10.3390/pharmaceutics15041195 (PMC10146622; doi:10.3390/pharmaceutics15041195)

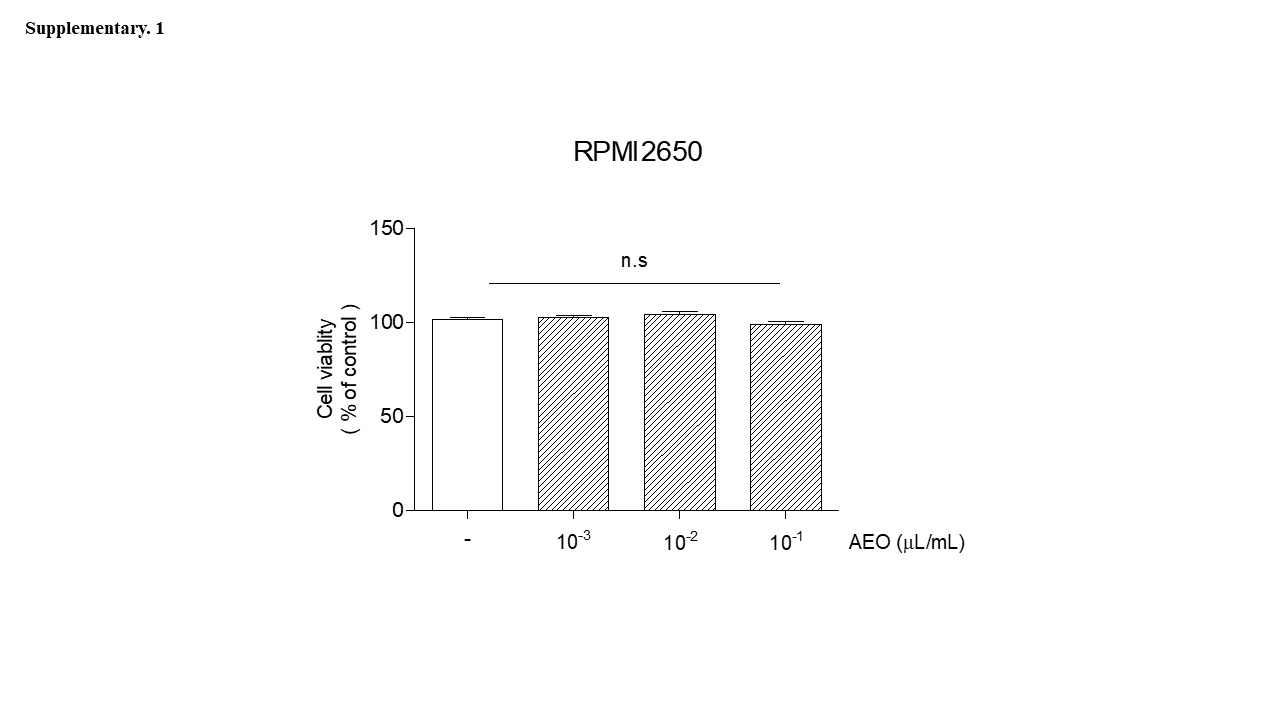

Supplement: Supplementary file 1 [file pharmaceutics-15-01195-s001.zip › Supplementary. 1.png]
